# Thyroid Cancer after Chornobyl: Increased Risk Persists Two Decades after Radioiodine Exposure

**DOI:** 10.1289/ehp.119-a306a

**Published:** 2011-07-01

**Authors:** Valerie J. Brown

**Affiliations:** Valerie J. Brown, based in Oregon, has written for *EHP* since 1996. In 2009 she won a Society of Environmental Journalists’ Outstanding Explanatory Reporting award for her writing on epigenetics.

During the 1986 Chornobyl nuclear accident, the then-Soviet republic of Ukraine was hit hard with iodine-131 fallout. Since then, numerous studies have demonstrated a relationship between I-131 exposure from Chornobyl and thyroid cancer risk. Much of the published research, however, has relied on grouped radiation dose estimates rather than individual estimates of radiation exposure. Now a new study using measurement-based individual dose estimates has shown the risk of developing thyroid cancer after I-131 exposure persists two decades later [*EHP* 119(7):933–939; Brenner et al.].

The U.S. and Ukrainian authors studied 12,514 individuals who in 1986 were under 18 years of age and living in one of three Ukrainian oblasts (states) contaminated with I-131 fallout from Chornobyl. The individuals’ I-131 exposure from the Chornobyl accident had been estimated using individual radioactivity measurements taken within two months of the accident, interview data, and ecological models of the fallout pattern. The study participants also were screened for thyroid cancer and other thyroid diseases a total of four times between 1998 and 2007. The current study did not include people who had been diagnosed with thyroid cancer during the first screening examination (1998–2000).

**Figure:**
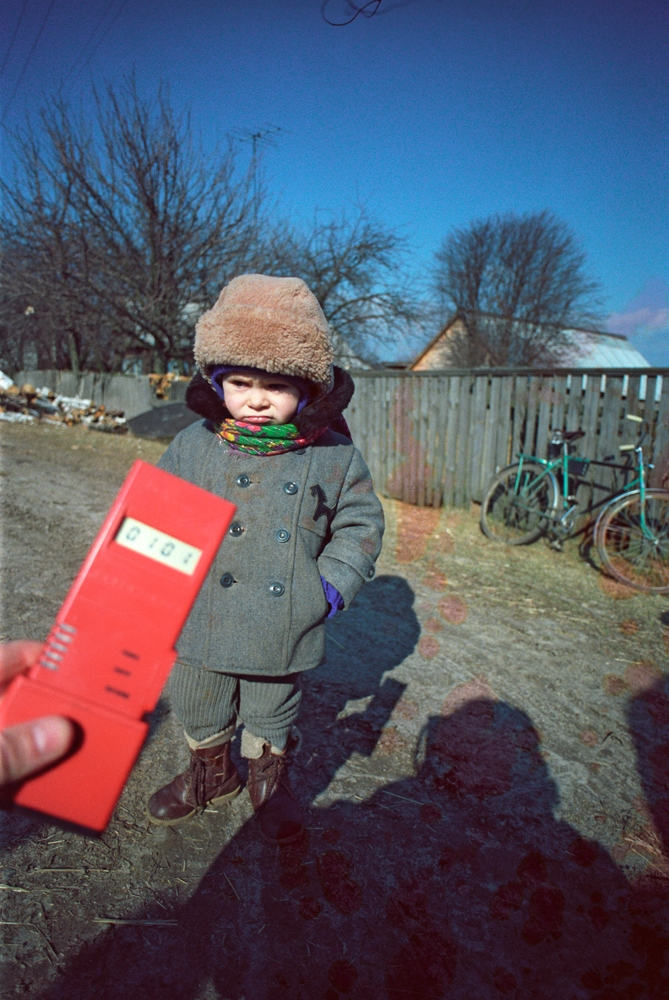
Exceedingly high radiation levels are measured in a village in Zhytomyr Oblast six months after the Chornobyl accident. © Igor Kostin/Sygma/Corbis

Sixty-five people in the cohort were diagnosed with histopathologically confirmed thyroid cancer after the first screening examination. Statistical analysis indicated the dose response was linear and that the estimated risk of thyroid cancer almost doubled for every Gray (a unit of absorbed radiation) of exposure. There was no reduction in the estimated radiation risk over time. Estimated risk varied significantly by oblast of residence. It also rose with younger age at exposure, and females were at a slightly increased risk over males, as were people with benign thyroid conditions such as diffuse goiter. However, none of these differences were statistically significant.

An earlier analysis of pooled data on external irradiation and thyroid cancer in other populations suggested the increased risk associated with radiation peaked 15–19 years after exposure but was still apparent 40 years later. It is unknown whether long-term effects of I-131 on thyroid cancer risk follow a similar pattern. The study’s comprehensive and detailed analyses strengthen existing evidence relating I-131 exposure and thyroid cancer risk; additional followup of this cohort is necessary to more accurately describe excess risk by time since exposure.

